# Diagnostic Value of Peripheral Blood Smear Tests in Identifying Candida Infections in a Tertiary Care Setting: A Case Series

**DOI:** 10.7759/cureus.83129

**Published:** 2025-04-28

**Authors:** Malvika Gaur, Tushar Sehgal, Ginni Bharti, Hemapriya J Babu, Immaculata Xess

**Affiliations:** 1 Laboratory Medicine, All India Institute of Medical Sciences, New Delhi, New Delhi, IND; 2 Microbiology, All India Institute of Medical Sciences, New Delhi, New Delhi, IND

**Keywords:** blood culture, candida, galactomannan, invasive fungal infections, peripheral blood film

## Abstract

Invasive fungal infections (IFIs) are a significant cause of morbidity and mortality in hospitalized patients, particularly among neonates and immunocompromised individuals. *Candida* species are one of the most commonly associated pathogens associated with IFIs. We present a case series of four patients with IFIs. All cases underwent peripheral blood film (PBF) examination, blood culture, and serum galactomannan testing. Budding yeasts were found in the PBF of three out of four cases. The findings were confirmed by culture. While blood culture remains the gold standard for diagnosing IFIs, the PBF test demonstrated its value as a rapid and cost-effective initial screening tool, especially in resource-limited settings. PBF tests can offer valuable preliminary insights, facilitating further diagnostic evaluation and early initiation of antifungal therapy. These findings underscore the potential utility of PBF tests in the early detection and management of IFIs.

## Introduction

Invasive fungal infections (IFIs) are a significant contributor to morbidity and mortality among hospitalized patients, particularly those with compromised immune function. Globally, the incidence of IFIs is rising, estimated at approximately six cases per 100,000 individuals annually [1]. *Candida *species are one of the most frequently implicated pathogens, especially among critically ill patients in intensive care units (ICUs), neonates, trauma patients, and the immunocompromised [2, 3]. An Indian study identified *Candida *species as the leading cause of bloodstream infections in ICUs, with *Candida tropicalis*, *Candida parapsilosis*, *Candida auris*, *Candida albicans*, and *Candida glabrata* being the most commonly isolated strains [4].

Invasive candidiasis (IC) accounts for roughly 9% of hospital-acquired bloodstream infections in the United States, ranking among the most common pathogens in medical, surgical, and ICU environments [5]. Likewise, Indian data have revealed a growing prevalence of non-albicans *Candida *species, such as *Candida tropicalis*, *Candida parapsilosis*, *Candida auris*, and *Candida glabrata*, particularly in ICU settings, possibly driven by widespread fluconazole use [6]. In a multicentric observational study conducted at 27 Indian ICUs, among 1,400 ICU-acquired candidemia cases, 65.2 % were adult. The important finding of the study was the vast spectrum of agents (31 *Candida *species) causing candidemia and a high rate of isolation of *Candida tropicalis* (41.6 %). Azole and multidrug resistance were seen in 11.8% and 1.9% of isolates. Public sector hospitals reported a significantly higher presence of the relatively resistant *Candida auris* and *Candida rugosa*. The 30-day crude and attributable mortality rates of candidemia patients were 44.7% and 19.6 %, respectively. The various significant independent predictors of mortality included admission to a public sector hospital, APACHE II score (short for Acute Physiology and Chronic Health Evaluation II) at admission, underlying renal failure, central venous catheterization, and steroid therapy [7].

Although blood culture remains the gold standard for diagnosing IFIs, peripheral blood film (PBF) examination is an often underutilized yet potentially valuable screening method. Identifying intracytoplasmic budding yeast cells within neutrophils or monocytes during PBF examination can indicate fungal infection early [1, 8-11]. While PBF examination is less sensitive than blood cultures, it may offer more rapid preliminary evidence, especially in severely ill patients who require immediate intervention [12]. However, interpreting PBF test results can be challenging in cases of low fungal load, emphasizing the need for meticulous examination and clinical awareness [13].

## Case presentation

Table 1 shows the clinical details of all the cases.

**Table 1:** Clinical details of all the cases

Case	Age	Gender	Predisposing condition	Outcome
1	10 days	Male	Preterm, low birth weight, post-surgical intestinal atresia	Died
2	40 years	Female	Post-surgery for adenocarcinoma of the colon	Responded to antifungal treatment
3	49 years	Male	Hepatitis C, recurrent ascites	Died
4	16 days	Male	Low birth weight	Died

Table 2 shows the results of all laboratory investigations.

**Table 2:** Results of all laboratory investigations WBC: white blood cell

Case	Age	Gender	Hemoglobin (g/dL)	Normal reference range of hemoglobin (g/dL)	WBC (10^9/L)	Normal reference range of WBC (10^9/L)	Platelet (10^9/L)	Normal reference range of Platelet (10^9/L)	Yeast form in PBF	Blood/swab Culture	Serum galactomannan
1	10 days	Male	9.5	11-17.3	15.5	3.1-21.6	21	152-472	Detected	Positive for *Candida albicans*	Positive
2	40 years	Female	10.5	12-15	19.9	4-11	80	150-450	Detected	Positive for *Candida albicans*	Positive
3	49 years	Male	8.9	13-17	11	4-11	155	150-450	Detected	Positive for *Candida glabrata*	Positive
4	16 days	Male	12.5	11-17.3	18.9	3.1-21.6	44	152-472	Detected	Positive for *Candida albicans*	Positive

**Figure 1:**
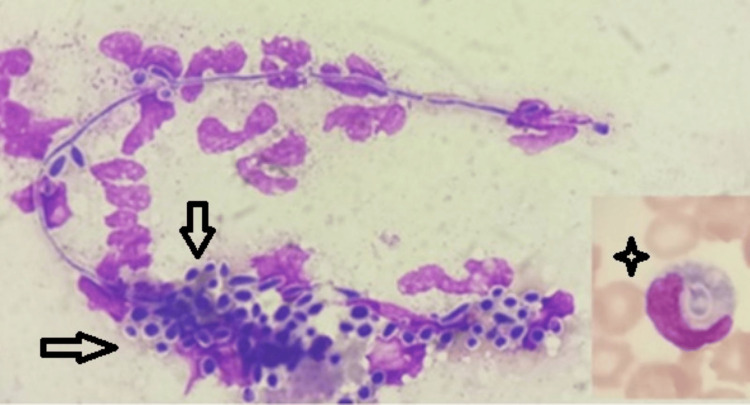
Peripheral blood film (PBF) examination shows pseudohyphae and intracellular and extracellular round-to-oval budding yeast cells (multiple arrows) engulfed by a monocyte (four-point star; Leishman and Giemsa stain, x1000)

A representative figure (Figure 1) shows the PBF examination with pseudohyphae and intracellular and extracellular round-to-oval budding yeast cells engulfed by a monocyte.

Case 1: a preterm neonate with intestinal atresia

A preterm, low-birth-weight 10-day-old male neonate was diagnosed with intestinal atresia and admitted to the neonatal intensive care unit (NICU) for further management. He required abdominal surgery for the correction of the intestinal obstruction. Initially, a complete blood count (CBC) and PBF examination showed normal results. However, thrombocytopenia was noted by day 5 of admission, prompting further investigation. On day 20, CBC revealed a white blood cell (WBC) count of 15.5 x 10^9/L (normal range, 3.1-21.6 x 10^9/L), hemoglobin of 9.5 g/dL (normal range, 11-17.3 g/dL), and a platelet count of 21 x 10^9/L (normal range, 152-472 x 10^9/L). On PBF examination, intracellular and extracellular round-to-oval budding yeast cells were observed, engulfed by both monocytes and neutrophils. A distinct zone of clearing was noted around the budding yeast cells, and pseudohyphae were also present. The neutrophils were increased, and toxic granules were demonstrated, accompanied by monocytosis. The findings suggest disseminated invasive fungal infection; a fungal culture was advised. Blood samples from the peripheral and central line catheters were sent for culture, and intravenous antifungal therapy was initiated the same day. Despite these interventions, blood culture results were negative. Follow-up blood samples collected on day 21 and day 22 showed a similar PBF picture, with persistent fungal forms engulfed by neutrophils and monocytes. However, by day 23, the fungal spores were no longer visible in the PBF examination, though thrombocytopenia persisted. A pharyngeal swab culture identified *Candida albicans*, and direct potassium hydroxide (KOH) preparation confirmed the presence of fungal elements. Additionally, the urine routine test revealed budding yeast forms (3+) (normal range, no yeast forms).

Case 2: an adult female with adenocarcinoma of the colon

A 40-year-old female patient with a medical history significant for adenocarcinoma of the colon was admitted to the ICU following multiple surgeries, including tumor resection and lymph node dissection. The patient required a colostomy bag and a central venous catheter for administering antibiotics and steroids as part of her postoperative management. On day 15 of hospitalization, the patient developed signs of systemic infection. Laboratory investigations revealed hemoglobin of 10.5 g/dL (normal range, 12-15 g/dL), WBC of 19.9 × 10^9/L (normal range, 4-11 × 10^9/L), and a platelet count of 80 × 10^9/L (normal range, 150-450 × 10^9/L). PBF examination showed fungal blastospores and pseudohyphae, indicating the presence of an invasive fungal infection. Additionally, neutrophilic leukocytosis and monocytosis were noted. The serum galactomannan test was positive. Blood samples were sent for culture, and empirical antifungal therapy with amphotericin B was promptly initiated. Subsequent blood culture results confirmed the presence of *Candida albicans*. Alongside the fungal infection, blood cultures isolated *Klebsiella pneumoniae*, a carbapenem-resistant gram-negative bacterium. Antibiotic susceptibility testing using the VITEK 2 system (bioMérieux Inc., Durham, NC, USA) revealed that the *Klebsiella pneumoniae* strain was sensitive only to colistin, necessitating the use of colistin. By the third day of antifungal therapy, a repeat PBF examination showed no fungal elements, indicating a successful initial response to treatment. A periodic acid-Schiff (PAS) stain performed on the initial blood smear further validated the diagnosis of invasive fungal infection by highlighting fungal structures. Despite the complexity of managing dual infections involving *Candida albicans* and multidrug-resistant *Klebsiella pneumoniae*, the patient’s condition improved with the combined antifungal and antibiotic therapy.

Case 3: an adult male with hepatitis C and recurrent ascites

A 49-year-old male patient with a longstanding history of hepatitis C virus infection was admitted to the hospital for the evaluation and management of recurrent ascites and jaundice. During his stay, the patient developed clinical features suggestive of systemic infection. PBF examination revealed intracellular budding fungi, raising the suspicion of IC. Further diagnostic workup included fungal culture, which confirmed the presence of *Candida glabrata*. The serum galactomannan test was also positive, indicating fungal involvement. CBC revealed hemoglobin of 8.9 g/dL (normal range, 13-17 g/dL), WBC of 11 × 10^9/L (normal range, 4-11 × 10^9/L), and a platelet count of 155 × 10^9/L (normal range, 150-450 × 10^9/L). The patient's pleural fluid culture also grew *Klebsiella *species, further complicating the clinical picture. C-reactive protein (CRP) levels were elevated at 87.5 mg/L (normal range, <3 mg/L), corroborating the presence of a significant inflammatory or infectious process. The patient's condition deteriorated rapidly despite initiating antifungal therapy and addressing the bacterial infection. The invasive fungal infection, combined with his underlying hepatitis C and bacterial co-infection, led to worsening clinical status. Unfortunately, the patient passed away shortly after the diagnosis of disseminated invasive fungal infection was made, highlighting the critical nature of fungal infections in immunocompromised individuals.

Case 4: a neonate with sepsis and low birth weight

A 16-day-old male neonate with low birth weight was brought to the hospital with clinical signs of sepsis. Initial investigations included a CBC and PBF examination. CBC revealed hemoglobin of 12.5 g/dL (normal range, 11-17.3 g/dL), WBC of 18.9 × 10^9/L (normal range, 3.1-21.6 × 10^9/L), and platelet count of 44 × 10^9/L (normal range, 152-472 × 10^9/L) during hospitalization. PBF examination revealed intracellular budding fungi, prompting further investigation for a suspected fungal infection. The blood culture identified *Candida albicans*, and the serum galactomannan test confirmed the diagnosis of IC. A urine culture also showed similar findings. Despite the initiation of aggressive antifungal therapy, the neonate’s condition continued to decline. The combined effects of invasive fungal infection and sepsis overwhelmed the neonate’s fragile physiology. Unfortunately, the neonate succumbed to sepsis shortly after the fungal infection was diagnosed, underscoring the critical importance of early detection and intervention in cases of neonatal fungal infections.

## Discussion

Our study emphasizes the potential role of PBF examination in diagnosing IC, a finding consistent with national and international studies. In our case series, intracellular budding yeast cells were detected on PBF examination, correlating with positive blood culture results and underscoring the value of PBF examinations for early identification. This observation aligns with previous Indian studies that successfully employed PBF examinations to diagnose *Candida *infections, particularly in ICU patients and neonates [4,10].

Hirai et al. [14] analyzed 36 cases in which the PBF examination showed candidemia. The most frequently isolated species was *Candida parapsilosis* (35.1 %), followed by *Candida albicans* (29.7 %). The overall mortality rate was 53.6 %. The time from the discovery of yeast-like pathogens using PBF to death ranged from a few hours to 93 days (median 19 days) [14]. Similarly, Kim et al. [15] observed that budding yeast cells within neutrophils on PBF could be an early indicator of candidemia. In our findings, *Candida albicans* was the most frequently identified species in both PBF and blood culture, consistent with prior Indian studies reporting *Candida albicans* as the predominant pathogen in hospitalized patients [6].

Timely diagnosis and initiation of antifungal therapy are critical, particularly for neonates and critically ill individuals. Despite treatment, IC is associated with high mortality rates; some studies report mortality of up to 55% in adults and 80% in neonates with *Candida* infections [16, 17]. Early detection is essential, and PBF examination can be a valuable adjunct to other diagnostic approaches in improving patient outcomes.

Although PBF examination has limitations, such as lower sensitivity and dependence on the fungal load, it remains a rapid, non-invasive screening tool. When combined with clinical judgment, blood cultures, and fungal antigen tests, careful examination of PBF can significantly contribute to effectively managing IFIs.

## Conclusions

PBF examinations can be a valuable tool for the early detection of IFIs, especially *Candida*, particularly in neonates and critically ill adults. Although its sensitivity is lower than that of blood cultures, PBF examinations can offer important diagnostic insights when used alongside other methods, such as blood cultures and fungal antigen testing. More extensive studies are needed to further validate the effectiveness of PBF examinations in diagnosing and managing IFIs, especially in high-risk patient groups.
